# Comment on “Confined placental mosaicism is a diagnostic pitfall in dystrophinopathies: a clinical report”

**DOI:** 10.1038/s41431-024-01723-7

**Published:** 2024-11-07

**Authors:** Diane Van Opstal, Brigitte H. W. Faas

**Affiliations:** 1 Department of Clinical Genetics, Erasmus MC, University Medical Center, Rotterdam, the Netherlands; 2 Department of Human Genetics, Radboud university medical center, Nijmegen, The Netherlands

**Keywords:** Cytogenetics, Mosaicism

Dear editor:

With considerable interest we’ve read the brief communication of Sabbagh et al. [1], recently published in this journal.

In their paper, the authors report on the presence of a mosaic intragenic deletion in the Duchenne muscular dystrophy (DMD) gene, detected by chromosomal microarray (CMA) analysis, and validated by MLPA analysis, in DNA isolated from a chorionic villus (CV) sample. Because of the mosaic nature of the deletion in the CV, DNA isolated from amniotic fluid was studied subsequently, to determine whether the deletion was also present in the foetus itself. No deletion was detected and a healthy boy was born. In their discussion, Sabbagh et al. conclude that their case “*emphasizes the importance of conducting amniocentesis following detection of mosaicism for single gene CNVs on chorionic villi, in order to preclude confined placental mosaicism (CPM)*”. Even though we do agree with the authors that this CPM is an interesting finding, and mosaicism detected in CV samples should be interpreted with care, we would like to comment on the method they used for sample preparation and on the above referred statement.

CV consist of two cell-layers, with a different embryonic origin, being the syncytio- and cytotrophoblast (CTB), derived from the trophoblast of the blastocyst, and the mesenchymal core (MC), derived from the inner cell mass (ICM) of the blastocyst (supplemental Figure 1). CPM is a well-known phenomenon that refers to the presence of genetically abnormal cells only present in the CV and not in the foetus, resulting from post-zygotic cell division errors. Based on the affected cell lineage, CPM can be categorised into CPM type I, with the genetic abnormality only present in the CTB, CPM type II, with the abnormality only present in the MC, and CPM type III, with the abnormality in both cell-layers [2, 3]. Cells from the MC cell-layer are more related to the foetus due to their common embryonic origin, the ICM (supplemental Figure 1), and the genotype of these cells is more representative for the actual foetal genotype than the genotype of cells from the CTB layer. Therefore, in the cytogenetic society, it is common practice to dissociate both cell layers [4] and to analyse their genetic constitution separately. Originally this was done by preparing short-term cultures (STC) of the CTB and long-term cultures (LTC) of the MC. If a chromosome aberration is found to be restricted to the CTB only (with normal MC), the diagnosis of CPM type I with a normal foetus is by far the most likely and follow-up investigations in amniotic fluid can be ommitted [5, 6]. Only a chromosomally abnormal MC needs a secondary amniocentesis for verification of the foetal karyotype. The original Specific Constitutional Cytogenetic Guidelines [7] stated that *“If an initial cytogenetic diagnosis is made on short-term preparations, a long term culture should be available for confirmation, in order to minimise problems of interpretation*” and “*Analysis solely on short-term incubation preparations is not recommended”*. The updated version of these guidelines [8] also recommend to dissociate the cell layers, in order to “*allow for mosaicism exclusion when needed”*. To minimize work load, nowadays in cytogenetic diagnostic laboratories it is common practice to only analyse DNA from the MC (not the CTB). By doing so, CPM type I, accounting for ~40% of all CPMs [6], will not be noticed but still CPM types II and III, that require follow-up testing in amniotic fluid, will be revealed.

For their analysis, Sabbagh et al. used DNA extracted from an uncultured, undigested CV sample, and thus analysed DNA from a mixture of cells from the CTB and the MC. If mosaicism is detected in such a mixture it is unknown which cell layer is affected and follow-up investigations in amniotic fluid are indicated to verify the foetal karyotype. This leads to unnecessary secondary invasive procedures in all cases of CPM type I, causing stress and a delay of a prenatal diagnosis.

As in all genetic fields, also in prenatal cytogenetic diagnosis, there is a shift from conventional cytogenetic analyses towards more molecular analyses. We certainly encourage and embrace this *molecularization*, and notice that the introduction of such molecular techniques goes along with a more pronounced collaboration with molecular laboratories in prenatal genetic diagnostics. But historically, the involvement of molecular laboratories in prenatal diagnostics has mainly been restricted to the analysis of familial variants, for which dissociation of the cell-layers is less crucial. Therefore, these laboratories often use DNA isolated from the total, undissociated CV for prenatal diagnosis. From routine cytogenetic studies focussing on microscopically visible chromosome aberrations, it is known that CPM affects 1-2% of all CV samples [9]. Percentages including submicroscopic Copy Number Variations (CNVs) vary and increase up to 4% [10]. When analysing DNA from undissociated CV, the detection of mosaicism should warrant confirmatory studies in amniotic fluid in order to differentiate between CPM and generalized mosaicism also affecting the foetus. We thus do agree with the statement of the authors that “*From a broader level, this report emphasizes the benefit of amniocentesis following detection of placental mosaicism for chromosomal rearrangements in order to reasonably rule out CPM and prevent unnecessary medical termination of pregnancy*”. However, we strongly feel there should be an additional message in the paper, being that the risk of detecting mosaicism caused by CPM type 1 should be avoided as much as possible, since it causes stress and delays a final prenatal diagnosis. This can simply be achieved by dissociating the CTB and MC cell-layers, as recommended in the European Guidelines for Constitutional Cytogenomic Analysis [8], and subsequently analysing DNA isolated from the MC only (or at least separately), instead of analysing DNA isolated from the complete, undissociated CV.

## Supplementary information

Supplemental Figure 1

Legend to supplemental Figure
