# Supplementary figures and images for: Comment on “Confined placental mosaicism is a diagnostic pitfall in dystrophinopathies: a clinical report”
Source: Eur J Hum Genet. 2024 Nov 7;34(1):3–4. doi: 10.1038/s41431-024-01723-7 (PMC12816563; doi:10.1038/s41431-024-01723-7)

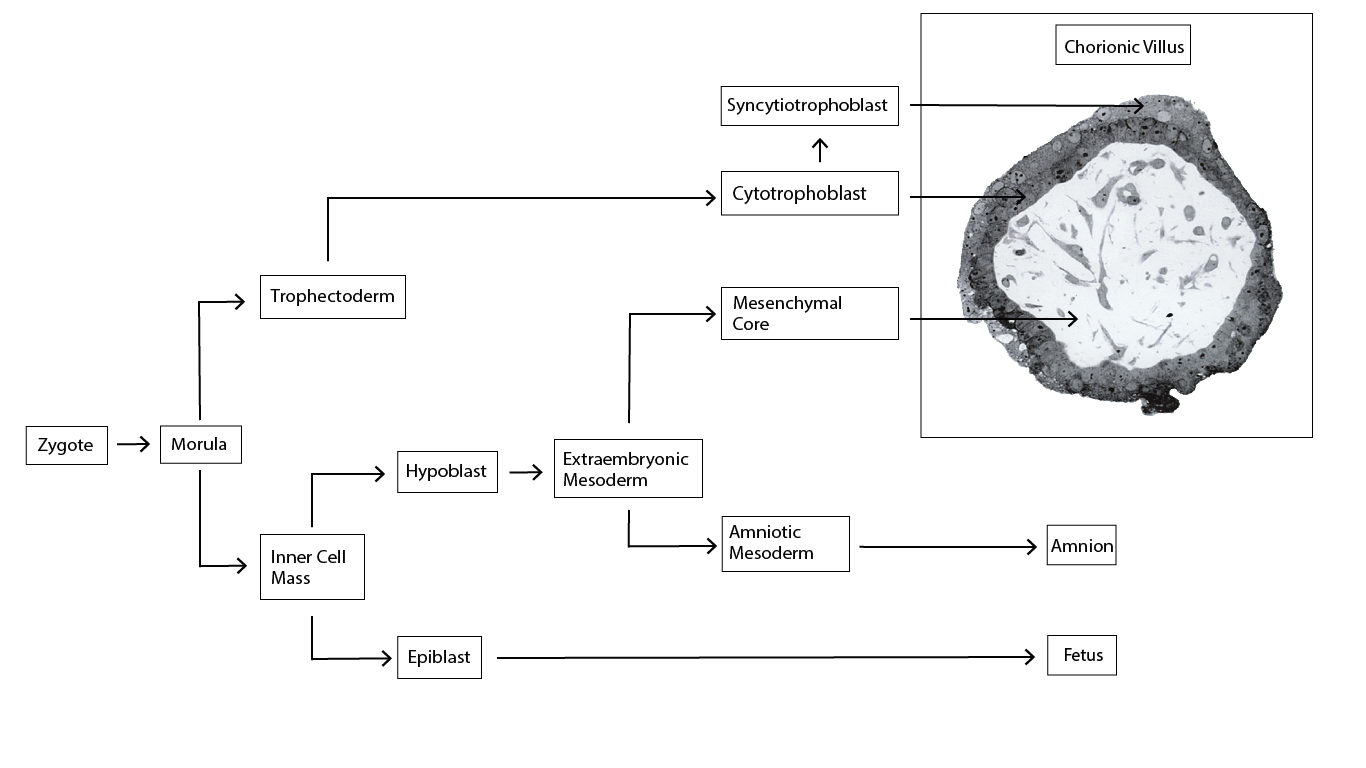

Supplement: Supplementary file 1 — Supplemental Figure 1 [file 41431_2024_1723_MOESM1_ESM.jpg]
