# Supplementary material for: Comment on “Confined placental mosaicism is a diagnostic pitfall in dystrophinopathies: a clinical report”
Source: Eur J Hum Genet. 2024 Nov 7;34(1):3–4. doi: 10.1038/s41431-024-01723-7 (PMC12816563; doi:10.1038/s41431-024-01723-7)
Supplement: Supplementary file 2 — Legend to supplemental Figure [file 41431_2024_1723_MOESM2_ESM.docx]

**Legend to supplemental Figure:**

Simplified diagram showing the early embryonic development and the origin of the cytotrophoblast and mesenchymal core of chorionic villi.
